# Hemodynamic support in the early phase of septic shock: a review of challenges and unanswered questions

**DOI:** 10.1186/s13613-018-0449-8

**Published:** 2018-10-29

**Authors:** Olivier Lesur, Eugénie Delile, Pierre Asfar, Peter Radermacher

**Affiliations:** 1 Division of Intensive Care Units, Department of Medicine, Faculté de Médecine et des Sciences de la Santé, Centre de Recherche du CHUS, Université de Sherbrooke, Sherbrooke, QC Canada; 2 Département de Médecine Intensive-Réanimation, Centre Hospitalier Universitaire, Université d’Angers, Angers, France; 3 Institut für Anästhesiologische Pathophysiologie und Verfahrensentwicklung, Universitätsklinikum, Ulm, Germany

**Keywords:** Sepsis, Septic shock, Hemodynamic support, Fluid resuscitation, Mean arterial pressure, Microcirculation, Vasopressor(s), Vasoactive drugs, Catecholamines, Decatecholaminization, Metabolic stress

## Abstract

**Background:**

Improving sepsis support is one of the three pillars of a 2017 resolution according to the World Health Organization (WHO). Septic shock is indeed a burden issue in the intensive care units. Hemodynamic stabilization is a cornerstone element in the bundle of supportive treatments recommended in the Surviving Sepsis Campaign (SSC) consecutive biannual reports.

**Main body:**

The “Pandera’s box” of septic shock hemodynamics is an eternal debate, however, with permanent contentious issues. Fluid resuscitation is a prerequisite intervention for sepsis rescue, but selection, modalities, dosage as well as duration are subject to discussion while too much fluid is associated with worsen outcome, vasopressors often need to be early introduced in addition, and catecholamines have long been recommended first in the management of septic shock. However, not all patients respond positively and controversy surrounding the efficacy-to-safety profile of catecholamines has come out. Preservation of the macrocirculation through a “best” mean arterial pressure target is the actual priority but is still contentious. Microcirculation recruitment is a novel goal to be achieved but is claiming more knowledge and monitoring standardization. Protection of the cardio-renal axis, which is prevalently injured during septic shock, is also an unavoidable objective. Several promising alternative or additive drug supporting avenues are emerging, trending toward catecholamine’s sparing or even “decatecholaminization.” Topics to be specifically addressed in this review are: (1) mean arterial pressure targeting, (2) fluid resuscitation, and (3) hemodynamic drug support.

**Conclusion:**

Improving assessment and means for rescuing hemodynamics in early septic shock is still a work in progress. Indeed, the bigger the unresolved questions, the lower the quality of evidence.

## Background

Sepsis is a leading cause of mortality, similar to that reported from acute myocardial infarction and lung or breast cancers in the USA [1]. This and other evidence prompted a WHO resolution in 2017 highlighting a crucial need for better recognition, assessment, and support in the near future [2]. Septic shock with multiple organ failure (i.e., the catastrophic phenotype of sepsis) represents over 50% of intensive care unit diagnostic profiles worldwide [1].

Despite advances in earlier recognition and a more effective management yielding a significant reduction in mortality rates, septic shock remains nonetheless a worrisome health care issue. The latest “Surviving Sepsis Campaign” (SSC) guidelines in 2016 were recently updated mid-2018 (supported by the SCCM and ESICM). Both 2016 and 2018 above guidelines have served as reference for the current recommendations [3, 4].

This review aims to focus on the hemodynamic support in early septic shock. Three essential topics have been selected as pillar elements and are discussed: (1) targets of hemodynamic stabilization, (2) fluid resuscitation, and (3) pharmacological hemodynamic support. Recommendations, gaps and controversies, ongoing research, and unanswered questions are exposed. With specific regards to the novel 2018 SSC update and its hemodynamic bundle of recommendations, a major modification was the period of time allotted to reach the threshold of 65 mmHg of MAP and mandates to administer the first 30 mL/kg fluid resuscitation within the 1st hour of admission (“hour-1 bundle”) and to introduce vasoactive agents (mainly norepinephrine—NE) sooner if this macro-circulatory goal is not achieved or not sustainably stable.

## Methods

Database selection, time window, and primary search terms (MeSH) used in the present review are detailed as followed.

A search strategy on MEDLINE and PubMed was operated, looking to and prioritizing randomized clinical trials (RCTs), systematic reviews, and meta-analyses (when existing) in articles published from 2013 to mid-2018. This exhaustive review focuses on sepsis hemodynamic support, which is a wide source of debate, and was starting on the former “Surviving Sepsis Campaign (SSC) Guidelines 2016,” recently updated in 2018 (supported by both SCCM and ESICM) as a central thread.

Primary search terms used (MeSH) were: sepsis, severe sepsis, septic shock, circulatory shock, distributive shock, shock, fluid resuscitation, mean arterial pressure, perfusion pressure, microcirculation, vasopressor(s), epinephrine, norepinephrine, dobutamine, decatecholaminization, beta-blocker(s), levosimendan, selepressin, arginine vasopressin, angiotensin, metabolic stress, and immunomodulation.

The highest level of evidence was used for RCTs and meta-analyses when available, using a PICO framework strategy.

Excluded were articles with data of patients under 18 years old and those relating to starch use, the latter being mostly eradicated from modern practice in this setting. The impact of the recently published Sepsis-3 definitions [5] was also not specifically explored.

## Which mean arterial pressure (MAP) target to stabilize the macrocirculation?

### Prognosis

Although the SSC 2016 recommends a MAP ≥ 65 mmHg during initial resuscitation (grade 1 B: strong recommendation, moderate level of evidence) [3], there is no precise evidence-based target determined to date. These guidelines suggest that the optimal MAP should be individualized and may be higher in selected patients such as those with atherosclerosis or previous hypertension [3]. In younger patients, a lower target may be acceptable.

The time spent below different threshold values of MAP during the first days has been analyzed and correlated with survival and organ dysfunction in two similarly designed retrospective studies using MAP recordings. The best MAP threshold was 65 mmHg, and the time spent under this value was positively correlated with mortality rate [6, 7].

A large prospective observational study (FINNAKI) [8] identified 423 patients with severe sepsis and showed that those with progression of acute kidney injury (AKI) within the first 5 days of ICU admission (36.2%) had a lower time-adjusted MAP than those without progression [9]. The best time-adjusted MAP value for predicting AKI progression was 73 mmHg. However, these data were not adjusted for disease severity. A retrospective analysis of health records of 8782 septic patients in the USA found increased mortality and AKI risks with time elapsed with average MAP below 85 mmHg [10].

In daily clinical practice, the actual objectified MAP level is often higher than the recommended target. This difference is also observed in all large prospective randomized controlled trials. Indeed, MAP was measured at 80 mmHg in three recent major clinical randomized trials aiming at comparing vasoactive drugs in patients with septic shock after 24 h of treatment (CATS, VASST, SOAP) [11–13]. Another study (SEPSISPAM) suggests that a MAP target of 65 mmHg is usually sufficient in patients with septic shock. However, a higher MAP level (around 75–85 mmHg) may prevent the occurrence of AKI in patients with chronic arterial hypertension [14]. Of note, patients with a high MAP target received significantly more norepinephrine (NE) and for a longer duration, while experiencing more cardiovascular side effects, especially new more onset of atrial fibrillation.

Given the aforementioned results, the SSC 2016 and 2018 Guidelines [3, 4] as well as the ESICM recommendations suggest targeting MAP to or over 65 mmHg for the initial resuscitation and to individualize MAP according to the patient’s comorbidities.

### Rationale for a “best MAP,” autoregulation…and microcirculation

In light of the above, MAP is commonly considered as a surrogate of global perfusion pressure, although several essential physiological particularities should be retained. Indeed, a better understanding of the autoregulatory mechanisms and microcirculatory regulation during sepsis is needed to rationally address this question. Furthermore, increasing MAP levels often (or always) imply increasing vasopressor load, raising the issue of vasopressor side effects, in addition to their action on MAP.

Autoregulation is the ability of an organ to maintain a constant blood flow entering the organ, irrespective of perfusion pressure, within a range of values called «autoregulation zone». Below this autoregulation threshold, the blood flow in the organ is directly dependent on perfusion pressure. Autoregulation is important in the brain [15], heart [16], and kidney [17], with varying autoregulation threshold values depending on the auto-regulated organ [16]. The kidney has the highest autoregulation threshold and may be considered as the first resuscitation objective, with regards to the potential impacts on the outcome [18]. Autoregulation thresholds differ with patient age and associated comorbidities (chronic hypertension). While autoregulation is a well-established key factor in acute stroke, it is still unknown whether it is maintained during sepsis and whether a traditional threshold remains unchanged [19].

Finally, perfusion pressure should not be regarded as being equivalent to MAP. Organ perfusion pressure is equal to the difference of the pressure in the artery entering the organ (usually approximated by MAP) minus the organ venous pressure. The importance of venous pressure has been shown, particularly in the kidney [20], and the relationship between a deficit of renal perfusion pressure and the risk of AKI has been reported in septic shock [21].

In addition, sepsis is associated with alterations in microcirculation characterized by increased endothelial permeability, leukocyte adhesion, and blood flow heterogeneity leading to tissue hypoxia [22, 23]. Microcirculatory blood flow may be independent from systemic hemodynamics [24]. Consequently, when systemic hemodynamic objectives (in particular MAP target) are achieved, microcirculation abnormalities may persist [23]. Hence, increasing MAP above 65 mmHg may not change microvascular perfusion. Thus, while adjusting hemodynamic objectives at the second phase of septic shock (when patients are “hemodynamically stable”) is unlikely to improve installed microcirculation impairment, an early intervention with high MAP levels may prevent the onset of microcirculatory dysfunction [25–30] (Table 1). However, more knowledge and monitoring standardization are requested to secure microcirculation assessment and related support. Two trials are currently ongoing with a peripheral or targeted tissue perfusion-guided primary objective (NCT01397474, NCT02579525).

**Table 1:** Prospective studies with MAP titration and peripheral (microcirculatory) or targeted tissue/organ perfusion assessment in septic shock

Authors [ref.]	No. of patients (*n*)	Design of MAP titration in mmHg (time at each step, min)	Main results
Ledoux et al. [25]	10	65, 75, 85 mmHg (**105**)	CI ↑ Arterial lactates, gastric intra-mucosal-arterial P_CO2_ difference, skin microcirculatory blood flow (skin capillary blood flow and red blood cell velocity), urine output: ns
Bourgoin et al. [26]	2 × 14	MAP 65 versus 85 mmHg (**240**) comparison of two groups	CI ↑ Arterial lactates, VO_2_, and renal function: ns
Deruddre et al. [27]	11	65, 75, 85 mmHg (**120**)	65–75 mmHg: urine output ↑, RRI ↓ 75–85 mmHg: urine output, RRI: ns Creatinine clearance: ns
Jhanji et al. [28]	16	60, 70, 80, 90 mmHg (**45**)	DO_2_, cutaneous PtO_2_, cutaneous microvascular red blood cell flux (laser Doppler flowmetry) ↑ Sublingual capillary MFI (SDF): ns
Dubin et al. [29]	20	65, 75, 85 mmHg (**30**)	CI, systemic vascular resistance, left and right ventricular stroke work indexes ↑ Arterial lactates, DO_2_, VO_2_, gastric intra-mucosal-arterial P_CO2_ difference, sublingual capillary MFI and percent of perfused capillaries (SDF imaging): ns
Thooft et al. [30]	13	65, 75, 85 mmHg (**30**)	CI, SvO_2_, StO_2_, sublingual perfused vessel density and MFI (SDF imaging) ↑ VO_2_: ns Arterial lactates ↓

*MAP* mean arterial pressure, *CI* cardiac index, *VO*_*2*_ oxygen consumption, *RRI* renal resistive index, *DO*_*2*_ oxygen delivery, *MFI* microvascular flow index, *SvO*_*2*_ mixed venous oxygen saturation, *StO*_*2*_ thenar muscle oxygen saturation using near-infrared spectroscopy (NIRS), *PtO*_2_ tissue oxygen pressure, *SDF* side-stream dark field

*ns* result not significant, ↑ increase, ↓ decrease

### Specific effect of high vasopressor load

Increasing the MAP target to high levels often requires high vasopressor doses. Norepinephrine (NE) is the most commonly used vasopressor in septic patients. It activates both alpha- and beta-adrenergic receptors and increases systemic vascular resistance (and thus left ventricle afterload); NE usually slightly increases cardiac output due to beta-adrenergic stimulation and its effect on venous return [31]. This venous effect of NE can also impact perfusion pressure, as outlined above [20]. In addition to the consequences of excessive vasoconstriction, other effects should also be taken into account when addressing the question of optimal vasopressor load. Sympathetic overstimulation (or adrenergic stress) can be associated with numerous harmful effects such as diastolic dysfunction, tachyarrhythmia, skeletal muscle damage (e.g., apoptosis), altered coagulation or endocrinological, immunological and metabolic disturbances [32].

## Fluid resuscitation: Should we do more or less, with what and when?

In the serial SSC bundles up to 2018, fluid resuscitation had been a recommended first-line cornerstone therapy to support or prevent induced cardiovascular dysfunction and for reducing in-hospital mortality in sepsis [3]. On admission obvious shortage of the effective circulatory volume in septic patients (e.g., decreased input, enhanced water loss, vascular leak or third space) is the essential premise underlying this recommendation. In this setting, fluid resuscitation must be initiated as soon as possible (ASAP) often at the emergency room, and definitely within 3–6 h, whether hypotension is obviously present or not, and to ensure optimal preload conditions for a hemodynamic homeostasis. Of note, from the original SSC 2004, derived from the protocol-based RCT (early goal-directed therapy: EGDT) which first reported an effective algorithmic approach for improving outcome in early sepsis [33], the “6 golden hours” were first abridged to “3 golden hours,” the earlier always being the better form of management, as highlighted by a recent retrospective cohort study [34]. Then, the emphasis has been placed on “ASAP” fluid resuscitation support (i.e., within the 1st hour of management, 30 mL/kg!) with further dynamic assessment enabling to identify patients who require more fluids and early introduction of vasopressors to reach a MAP target in the 2018 recommendations [4].

Indeed, even with differences in timing and previous intervention(s) before randomization, three successive RCTs (ProCESS, ARISE, ProMISe) [35–37] subsequently showed no benefit in primary mortality outcomes of an EGDT-like protocol-based approach, including lack of cost-effectiveness, such that new strategies are mandatory [38].

“*at least 30* *mL/kg of crystalloids within the first 3* *h…” (*strong recommendation, low quality of evidence, SSC 2016) [3] may be within the first hour! (SSC 2018) [4].

This “fixed” minimum fluid loading is actually recommended at this step (with or without vasopressor addition) if a minimum MAP of 65 mmHg is not achieved “rapidly” (SSC 2018) [4]. The early introduction of vasopressors, which is currently observed in many studies, is not disapproved and may have outcome benefits even more [39]. Of note, about 50% of septic patients in shock are non-responsive to fluids, only half patients included in the three above cited RCTs received 30 mL/kg in this time window, and the median fluid volume infused within the first 4 h before randomization in the recent VANISH trial was below 1.7 L [35–37, 40, 41]. Of course, clinical judgment is always the rule, and the evidence of pulmonary venous congestion, for example, should waive this fluid resuscitation practice.

*“..additional fluids afterward, guided by frequent reassessment of hemodynamic status…”* (best practice statement 2016) [3, 4].

Because “one size does not fit all,” personalized assessment is suggested after the initial fluid load, mandating identification and selection of responding patients who require more fluids.

The goals of initial resuscitation can be central venous pressure (CVP), MAP, urine output, central venous oxygen saturation (ScvO_2_), or blood lactates, although more dynamic variables than rigid static goals are suggested and proposed (e.g., pulse pressure variation, stoke volume variation, superior vena cava collapsibility, respiratory variation of inferior vena cava, end-expiratory occlusion test, or passive leg raising test). These still need further validation because prediction of fluid responsiveness is not a current practice worldwide [42].

Indeed, too much fluid is just as detrimental as too little and “*primum non nocere*.” An increased risk of death was demonstrated with > 5 L first day, as already raised in VASST, and positive fluid balance significantly associated with enhanced mortality as early as 12 h after onset of management [43, 44]. Whether this may be only a severity marker rather than a causal relationship remains to be proven, but a negative fluid balance at 72 h within a “deresuscitation” strategy is associated with lower mortality [45].

Anyway, more restrictive/conservative or “deresuscitation” fluid resuscitation strategies are currently under evaluation (ACTRN12616000006448, NCT02079402, NCT0247371).

*“crystalloids are to be selected in both above steps”* (strong recommendation, moderate quality of evidence) [3, 4].

The question of which crystalloid (pH balanced or not) should be preferred still remains an ongoing debate. A recent RCT, including over 15,000 patients cluster randomized in a multiple crossover trial, has challenged the use of balanced crystalloids versus normal saline (NS) in critically ill conditions [46]. Less adverse kidney events and a trend in 30-day mortality reduction were observed with balanced crystalloids, with numbers needed to treat (NNTs) of 91 and 125, respectively. However, no distinction between balanced crystalloids was mentioned and septic patients represented less than 15% of included subjects (*n* = 2336). In this latter subset, a gain in targeted outcomes in favor of balanced fluids was noted: e.g., more reductions of 30-day mortality and major renal or other events (weak recommendation, moderate quality of evidence) [46].

Several additional RCTs comparing NS versus balanced crystalloid solution are currently ongoing (NCT02875873, NCT03277677), and comparative investigations on outcomes in-between the balanced crystalloid solution portfolio (e.g., lactated Ringer vs. Plasma-Lyte A) should be mandated.

*“using albumin (Alb) in addition to crystalloids when a substantial amount of fluids is needed” (*weak recommendation, low quality of evidence) [3, 4].

A first subgroup analysis of septic shock patients in the SAFE study trended toward a reduction in mortality [47], while no difference in targeted mortality rates was observed in the ALBIOS as well as EARSS trials (never published!) [48, 49]. Trends toward reduced mortality in several small studies and in meta-analyses have been reported (Table 2) [50–53], although the latter suffer from differing designs, types of Alb (iso- vs. hyper-oncotic), and infusion modalities (Alb used as a resuscitation fluid vs. as a pleiotropic molecule [54]).

**Table 2:** Systematic reviews and meta-analyses on albumin use as a resuscitation fluid in sepsis/septic shock

Systematic reviews [ref.]	No. of patients (*n*)	No. of RCTs included (presented)	Intervention fluid therapy	Primary outcome	Results: albumin versus crystalloids	Comments
Bansal et al. [50]	6082	13 (6^†^)	Albumin, crystalloids [HES]	Mortality	*OR 0.9 (0.8–1.01)	2 RCTs including children and 1 case mix
				RRT need	?	7 RCTs with specific comparison HES versus crystalloids
Xu et al. [51]	5838	5	Albumin, crystalloids	All-cause mortality	** OR 0.88 (0.76–1.01) *p *= 0.08 severe sepsis OR 0.81 (0.67–0.97) *p *= 0.03 Septic shock	4 of 5 RCTs not entirely dedicated to septic patients
Patel et al. [52]	4190	16^†^	Albumin, crystalloids	All-cause mortality	RR 0.93 (0.86–1.01) *p *= 0.07	~ 10 RCTs not entirely dedicated to septic patients
Rochwerg [53]	1238^††^	14 (2)	Albumin, crystalloids	All-cause mortality	NMA 0.83 (0.65–1.04) estimate	Only 2 RCTs with direct comparison and one multicentric subgroup analysis encompassing more than 98%

*RCT*_*S*_ randomized control trials, *OR* odds ratio, *RR* relative risk, *HES* hydroxy ethyl starches, *NMA* nodal meta-analysis

*28- and 30-day mortality

**90-day mortality

^†^One EARSS from the reported conference proceedings

^††^Post hoc analyses: (1) ALBIOS trial patients (*n *= 1815) not included because Alb was not used as a resuscitation fluid; data incorporation did not affect the final results, (2) exclusion of data from the one trial encompassing less than 2% of patients did not affect the final results

In addition, given the raised potential adverse impact of high chloride fluid infusion, concerns as to variable chloride contents in different commercial Alb products have recently been reported [55], and it is noteworthy in this context that in turn increasing albuminemia may decrease pH due to a higher anion gap [56].

*“the frailty cardio*-*renal axis….”*

While there is currently no strong indication as to what constitutes a “better” fluid selection in improving morbidity and mortality rates in sepsis [3], there is increasing evidence since several decades that (1) patients receiving the largest fluid resuscitation were those with the worse outcome [43, 44], (2) adverse events and outcomes can occur as early as 12 h after sepsis onset when fluid resuscitation is sustained [44], and (3) sepsis-associated AKI is both common and costly (e.g., renal replacement therapy—RRT). With the exception of hydroxyethyl starches (HES) (including last generation), which are associated with more frequent and severe AKI and higher RRT needs [57], protocolized resuscitation does not appear to be an influencing factor, and balanced crystalloids have either marginally or never reduced the above outcomes to date [46, 56, 58–60].

## Hemodynamic drug support:… to be or not to be?

### Catecholamines

‘According to the most recent SSC 2016–2018 Guidelines [3] “*norepinephrine (NE) is recommended as the first*-*choice vasopressor (strong recommendation, moderate quality of evidence)”* because of its vasopressor and positive inotropic properties as well as its effect on venous return [61]. These guidelines also “*suggest epinephrine (E) to NE with the intent of raising MAP to target (weak recommendation, low quality of evidence)*.” E titrated to comparable systemic hemodynamic targets clearly results in more pronounced metabolic stress than NE [62], although to date, large RCTs have failed to show the superiority of NE alone [12] or in combination with dobutamine [11] in septic shock when compared to E. Dobutamine is frequently used as an inotropic drug, and accordingly, the SSC 2016–2018 Guidelines [3] suggest its use “*…in patients who show evidence of persistent hypoperfusion despite adequate fluid loading and the use of vasopressor agents*.” However, in contrast to the use of NE this rational only represents a “*weak recommendation with low quality of evidence*.” In fact, the data supporting the use of dobutamine are “*…primarily physiologic, with improved hemodynamics and some improvement in indices of perfusion….”* There are no RCT on the use of dobutamine alone, and, as mentioned above, NE in combination with dobutamine was similar to E with respect to overall outcome. Moreover, from a pharmacological point of view, the efficacy of dobutamine per se might be limited when used in combination with NE: in vitro, dobutamine is a weak β-adrenergic agonist when compared to NE [63], and a comparably lower activity of dobutamine than NE was demonstrated in healthy volunteers with respect to catecholamine-induced glucose and lactate metabolism [64]. This issue may assume particular importance in the context of the sepsis-related adrenoceptor desensitization, which is exacerbated by ongoing catecholamine treatment [65]. Furthermore, catecholamines exhibit marked immune-modulatory properties [66] and are known to profoundly affect energy, in particular glucose metabolism [67], and inhibit gastrointestinal peristalsis (for review: see [68, 69]). In addition, “*vasopressor load”* from high-dose catecholamine infusion rates has been found to be directly related to mortality regardless of the specific MAP achieved [70] due to catecholamine-induced cardiac toxicity [71]. Therefore, the concept of “*decatecholaminization”* has been put forward in the last decade [72, 73]. Several approaches have been tested, including arginine vasopressin (AVP) or its synthetic analogs, levosimendan, angiotensin II, as well as β-blockade (Table 3). The most abundant data available is on arginine vasopressin (AVP). Albeit “*not recommended as a first line vasopressor,*” the SSC 2016–2018 Guidelines [3] in fact “*suggest adding…vasopressin (up to 0.03 U/min)…to decrease NE dosage (weak recommendation, moderate quality of evidence)*.” This addition has “catecholamine-sparing” capacity [12] and was recently proven to lower risk of new onset atrial fibrillation in patients with distributive shock [74], which is per se a significant worsening factor of in-hospital stroke and mortality in sepsis [75]. Nevertheless, so far there has been no clear evidence from RCT that the “*decatecholaminization”* concept is really more efficient than the standard approach using NE. However, data from the VASST, ATHOS, and esmolol trials demonstrated its feasibility, safety and, moreover, suggested improved morbidity and mortality (see below).

**Table 3:** Hemodynamic drug support and RCTs in septic shock

Acronym	Studied drugs	Type of study	No. of patients (*n*)	Primary outcome	Main results	Authors [ref.]
VASST	AVP versus NE	RCT, double blind, multicenter	778 (396 vs. 382)	Mortality at day 28	No difference; significantly lower mortality in patients with NE < 15 µg/min	Russell et al. [12]
VASST (post hoc according to sepsis 3.0)	AVP versus NE	RCT, double blind, multicenter	375 (193 vs. 182)	Mortality at day 28	Significantly lower mortality in patients with lactate ≤ 2 mmol/L	Russell et al. [77]
VANISH	AVP versus NE (subsequently HCT versus placebo)	2 × 2 RCT, double blind, multicenter	409 (104 vs. 103 vs. 101 vs. 101)	Kidney failure-free days until day 28	No difference	Gordon et al. [41]
VANC	AVP versus NE	RCT, double blind, single center	300 (149 vs. 151)	Mortality and/or severe complications	Significantly less acute renal failure and atrial fibrillation	Hajjar et al. [78]
SEPSIS-ACT	Selepressin versus NE	RCT, double blind, multicenter	53 (32 vs. 21)	MAP > 65 mmHg without NE; NE dose	Significantly lower NE load, less net fluid intake, more ventilator-free days	Russell et al. [81]
LeoPARDS	Levosimendan versus standard treatment alone	RCT, double blind, multicenter	516 (259 vs. 257)	SOFA score up to day 28	No difference; higher incidence in supraventricular tachyarrhythmia	Gordon et al. [83]
ATHOS-3	Angiotensin II versus NE	RCT, double blind, multicenter	321 (163 vs. 158)	Target MAP > 75 mmHg at 3 h	Significantly more patients with target achieved; higher reduction in SOFA score at 48 h	Khanna et al. [86]
nn	Esmolol versus conventional treatment	Open label, RCT, single center	154 (77 vs. 77)	80 < heart rate < 95 over 96 h	Significantly lower mortality at day 28	Morelli et al. [88]

*RCTs* randomized clinical trials, *NE* norepinephrine, *AVP* arginine vasopressin, *HCT* hydrocortisone, *nn* no name

### Vasopressin (AVP) and analogs

Overall, the VASST trial more than 10 years ago did not find any outcome benefit for low-dose (0.01–0.03 U/min) AVP compared to NE [12]. However, in contrast to the underlying hypothesis that the more severe patients might benefit from this approach, the subgroup of patients with only moderate NE requirements (pre-defined as < 15 μg/min), i.e., those in whom weaning from NE was more frequent [76], presented significantly improved survival. Moreover, more patients died while still on NE in the NE group than in the AVP group. Interestingly, a post hoc analysis of the VASST database according to the Septic Shock 3.0 definition [5] showed that AVP lowered the mortality rate compared to NE in patients with lactate levels ≤ 2 mmol/L [77]. The VANISH trial, a 2 × 2 comparison of either AVP (up 0.06 U/min) or NE as initial vasopressor to maintain target MAP followed by hydrocortisone (HCT) or placebo, did not improve the number of kidney failure-free days, although the confidence interval did suggest a potential benefit for AVP [41]. Finally, the single-center VANCS trial showed that AVP (0.01–0.06 U/min) used as first-choice vasopressor reduced morbidity (in particular the incidence of acute renal failure and de novo atrial fibrillation) in vasoplegic patients post-cardiac surgery [78].

AVP non-selectively activates all vasopressin receptor subtypes and the oxytocin receptor, thus potentially resulting in undesirable side effects *(*e.g., water retention, platelet aggregation) other than the hemodynamic targets [79]. Therefore, more selective V_1_ agonists have been tested. While the use of terlipressin does not “*offer advantages over AVP”* [80] due to its long duration of action over several hours, the pilot SEPSIS-ACT trial of the new, short-acting selective V_1A_ receptor agonist selepressin reduced cumulative NE doses and net fluid balance, and it increased the number of ventilator-free days [81].

### Levosimendan

Many patients with sepsis develop cardiac dysfunction (“*septic cardiomyopathy”* [82]), which prompted the investigation of the “*calcium sensitizer”* levosimendan. The LeoPARDS trial comparing levosimendan (0.05–0.2 µg/kg/min, depending on rate-limiting side effects) and placebo in addition to standard treatment neither reduced sepsis-induced organ failure nor affected mortality or any other secondary outcome. Levosimendan was associated, however, with a higher incidence of supraventricular tachyarrhythmia [83].

### Angiotensin II

It has been known for decades that septic shock causes activation of the renin–angiotensin–aldosterone system [84], which leads to angiotensin II release [85]. The ATHOS-3 trial compared angiotensin II (1.25–40 ng/kg/min) or placebo to achieve a target MAP ≥ 75 mmHg in patients with vasodilatory shock receiving NE > 0.2 µg/kg/min [86]. This primary endpoint was reached in a significantly higher proportion of patients in the treatment versus the placebo arm (69.9 vs. 23.4%). While the number of serious adverse events and mortality at day 28 did not differ between the two groups, angiotensin II-treated patients exhibited a greater improvement in organ failure score(s) at 48 h.

### β-Blockade

At first glance, β-blockade appears to be counterintuitive in patients with vasodilatory shock depending on vasopressor therapy, i.e., catecholamine treatment to achieve target MAP. However, based on the similarity in hyperadrenergic response between patients with septic shock and those with cardiac disease [87], an open-label trial in patients with septic shock requiring continuous i.v. NE and presenting with a heart rate > 95/min after 24 h of ICU care investigated the infusion of the short-acting β-blocker esmolol titrated to maintain heart rate at 80–94/min for 96 h in addition to conventional treatment [88]. Esmolol treatment coincided with a lower area under curve for lactatemia and need of fluid resuscitation, and it was ultimately associated with a significantly lower mortality than in the conventional treatment group (49.4 vs. 80.5%).

Given the side effects of high-dose catecholamine treatment and the consequences of sympathetic overstimulation, new approaches based on the concept of “*decatecholaminization”* are being considered by the latest SSC Guidelines to treat sepsis-induced vasoplegia. On the other hand, angiotensin II and β-blockers—if used—should be handled with considerable caution and only in selected patients. New drug prospects for an optimized ventriculo-arterial coupling are currently under investigation [89].

### Hydrocortisone (HCT)

Albeit HCT is not a hemodynamic drug in the sense of direct vasopressor and/or inotropic activity, since its first use in small-sized trials in the late 1990s [90, 91], the existing RCT data unanimously showed that HCT allowed accelerated resolution of shock as defined by complete weaning from vasopressor support to achieve MAP targets [92, 93]. The hastened resolution of circulatory shock was referred to attenuation of the sepsis-induced hyper-inflammatory response, inhibition of the inducible isoform of the nitric oxide (NO) synthase, thereby attenuating excess NO release, and improved adrenergic receptor responsiveness [95]. Nevertheless, since overall outcome results were equivocal, inasmuch both improved survival [94] and unchanged survival [92, 93] were reported, the use of HCT remains a matter of debate. Accordingly, the SSC 2016–2018 Guidelines [3]—which could not take into the account the more recent ADRENAL [93] and APROCCHSS [94] trials—in fact suggest “*…i.v. hydrocortisone at a dose of 200* *mg per day”* only if adequate fluid resuscitation and vasopressor support do not allow restoring hemodynamic stability, however, as a weak recommendation with low quality of evidence. Clearly, HCT seems not to have any beneficial effect in the prevention of septic shock [96] and should be tapered down once resolution of shock is achieved [3]. Of note, in the context of “*decatecholaminization*,” HCT may assume particular importance: a post hoc analysis of the VASST data base demonstrated a significant interaction between AVP and HCT, inasmuch the un-protocolized use HCT was associated with attenuated mortality and morbidity in the AVP arm, whereas the opposite result was found in patients who did not receive HCT [97].

## Conclusion

Hemodynamic support in sepsis and septic shock is a perpetual work in progress.

Fluid resuscitation with crystalloids remains cornerstone of supportive therapy, “*the earlier the better*,” although “*too much is just as detrimental as too little*.” Targeted goals for fluid cannot be pre-established, and dynamic monitoring and personalization are mandatory. Actual and recommended MAP target is 65 mmHg but must be adapted according to patient comorbidities (i.e., chronic hypertension) and with the understanding that convergence toward macro-to-microcirculation perfusion synchrony is difficult to reach. Vasoactive and potentially inotropic catecholamines are still (and potentially urgently) recommended for pharmacological hemodynamic support, although additional supportive molecules (e.g., vasopressin, angiotensin II) and new agents/approaches tend toward a new paradigm of “decatecholaminization.”

## Unanswered questions

However, while knowledge is growing and has already provided improvements toward a better assessment and monitoring of hemodynamics in patients undergoing sepsis, unresolved questions are bigger than the quality evidence, “…a little bit does go a long way” in this instance! Several unanswered questions with regards to the recommended SSC 2018 Guidelines are summarized in Table 4.

**Table 4:** Hemodynamics in early septic shock

Main questions	Actual recommendations*	Unanswered questions
Which MAP targets to stabilize the macrocirculation?	MAP ≥ 65 mmHg	What is the best timing for MAP intervention in sepsis? and until when? Could “permissive hypotension” be considered as in the case of trauma? for which reason(s) and target(s)?
How much fluid resuscitation and when?	From “time of presentation” or “time zero,” 30 mL/kg at least within 1 h	Should we prioritize fixed minimum fluid resuscitation or dynamic personalized reassessment of circulation status?
Which fluid(s)?	Crystalloids	Beyond balanced versus unbalanced crystalloid fluid selection, should we prefer acetate- or lactate-buffered solutions?
How long?	After the initial 1-h interventions, further fluid administration needs patients’ assessment for responsiveness	What “gauge for a filled tank”?
Which vasoactive (± inotropic) drug(s)?	NE is recommended as a 1st choice vasopressor. AVP or E can be added to help reaching the target (i.e., MAP) and spare NE	Within a “hour-1 bundle” strategy, should we trade-off less fluids and more vasoactive drugs to vice versa?
When?	Dobutamine only if target not reached after adequate fluid loading and use of vasoactive drugs	Are vasopressor combinations able to reach high MAP levels without detrimental cardiac side effects?
	As early as during the initial fluid resuscitation period, to achieve the target MAP ≥ 65 mmHg ASAP	With NE as the currently recommended first-line vasopressor is “decatecholaminization” feasible and safe?

*MAP* mean arterial pressure, *NE* norepinephrine, *AVP* arginine vasopressin, *E* epinephrine, *ASAP* as soon as possible

*According to the Surviving Sepsis Campaign 2016 and the 2018 update (Refs [3, 4])

## Abbreviations

WHO: World Health Organization
SSC: Sepsis Surviving Campaign
RCT(s): randomized clinical trial(s)
PICO: patient, intervention, comparison, outcome
MAP: mean arterial pressure
AKI: acute kidney injury
ICU: intensive care unit
NE: norepinephrine
CI: cardiac index
VO_2_: oxygen consumption
DO_2_: oxygen delivery
MFI: microvascular flow index
SvO_2_: mixed venous oxygen saturation
StO_2_: tissue oxygen saturation
NIRS: near-infrared spectroscopy
Pt: tissue oxygen pressure
SDF: sidestream dark field
RRI: renal resistive index
ASAP: as soon as possible
CVP: central venous pressure
Scv: central venous oxygen saturation
NS: normal saline
NNT: number needed to treat
Alb: albumin
RTT: renal replacement therapy
HES: hydroxyethyl starches
OR: odds ratio
RR: relative risk
NMA: nodal meta-analysis
E: epinephrine
AVP: arginine vasopressin
HCT: hydrocortisone
NO: nitric oxide

## References

[1] Martin G, Mannino D, Eaton S (2003). The epidemiology of sepsis in the U-S from 1979 through 2000. N Engl J Med.

[2] Reinhart K, Daniels R, Kissoon N (2017). Recognizing sepsis as a global health priority—a WHO resolution. N Engl J Med.

[3] Rhodes A, Evans LE, Alhazzani W (2017). Surviving Sepsis Campaign: international guidelines for management of sepsis and septic shock: 2016. Intensive Care Med.

[4] Levy M, Evans LE, Rhodes A (2018). The Surviving Sepsis Campaign Bundle: 2018 update. Intensive Care Med.

[5] Singer M, Deutschman CS, Seymour CW (2016). The third international consensus definitions for sepsis and septic shock (sepsis-3). JAMA.

[6] Varpula M, Tallgren M, Saukkonen K (2005). Hemodynamic variables related to outcome in septic shock. Intensive Care Med.

[7] Dünser MW, Takala J, Ulmer H (2009). Arterial blood pressure during early sepsis and outcome. Intensive Care Med.

[8] Nisula S, Kaukonen K-M, Vaara ST, The FINNAKI Study Group (2013). Incidence, risk factors and 90-day mortality of patients with acute kidney injury in Finnish intensive care units: the FINNAKI study. Intensive Care Med.

[9] Poukkanen M, Wilkman E, Vaara ST, The FINNAKI Study Group (2013). Hemodynamic variables and progression of acute kidney injury in critically ill patients with severe sepsis: data from the prospective observational FINNAKI study. Crit Care Lond Engl.

[10] Maheshwari K, Nathanson BH, Munson SH (2018). The relationship between ICU hypotension and in-hospital mortality and morbidity in septic patients. Intensive Care Med.

[11] Annane D, Vignon P, Renault A, The CATS Study Group (2007). Norepinephrine plus dobutamine versus epinephrine alone for management of septic shock: a randomised trial. Lancet.

[12] Russell JA, Walley KR, Singer J, The VASST Investigators (2008). Vasopressin versus norepinephrine infusion in patients with septic shock. N Engl J Med.

[13] De Backer D, Biston P, Devriendt J, The SOAP II Investigators (2010). Comparison of dopamine and norepinephrine in the treatment of shock. N Engl J Med.

[14] Asfar P, Meziani F, Hamel J-F (2014). High versus low blood-pressure target in patients with septic shock. N Engl J Med.

[15] Strandgaard S, Olesen J, Skinhoj E (1973). Autoregulation of brain circulation in severe arterial hypertension. Br Med J.

[16] Berne RM (1964). Regulation of coronary blood flow. Physiol Rev.

[17] Cupples WA, Braam B (2007). Assessment of renal autoregulation. Am J Physiol Renal Physiol.

[18] Badin J, Boulain T, Ehrmann S (2011). Relation between mean arterial pressure and renal function in the early phase of shock: a prospective, explorative cohort study. Crit Care Lond Engl.

[19] Bellomo R, Wan L, May C (2008). Vasoactive drugs and acute kidney injury. Crit Care Med.

[20] Legrand M, Dupuis C, Simon C (2013). Association between systemic hemodynamics and septic acute kidney injury in critically ill patients: a retrospective observational study. Crit Care Lond Engl.

[21] Panwar R, Lanyon N, Davies AR (2013). Mean perfusion pressure deficit during the initial management of shock—an observational cohort study. J Crit Care.

[22] De Backer D, Donadello K, Taccone FS (2011). Microcirculatory alterations: potential mechanisms and implications for therapy. Ann Intensive Care.

[23] De Backer D, Creteur J, Preiser J-C (2002). Microvascular blood flow is altered in patients with sepsis. Am J Respir Crit Care Med.

[24] De Backer D, Ortiz JA, Salgado D (2010). Coupling microcirculation to systemic hemodynamics. Curr Opin Crit Care.

[25] LeDoux D, Astiz ME, Carpati CM (2000). Effects of perfusion pressure on tissue perfusion in septic shock. Crit Care Med.

[26] Bourgoin A, Leone M, Delmas A (2005). Increasing mean arterial pressure in patients with septic shock: effects on oxygen variables and renal function. Crit Care Med.

[27] Deruddre S, Cheisson G, Mazoit J-X (2007). Renal arterial resistance in septic shock: effects of increasing mean arterial pressure with norepinephrine on the renal resistive index assessed with Doppler ultrasonography. Intensive Care Med.

[28] Jhanji S, Stirling S, Patel N (2009). The effect of increasing doses of norepinephrine on tissue oxygenation and microvascular flow in patients with septic shock. Crit Care Med.

[29] Dubin A, Pozo MO, Casabella CA (2009). Increasing arterial blood pressure with norepinephrine does not improve microcirculatory blood flow: a prospective study. Crit Care Lond Engl.

[30] Thooft A, Favory R, Salgado DR (2011). Effects of changes in arterial pressure on organ perfusion during septic shock. Crit Care Lond Engl.

[31] Hamzaoui O, Georger J-F, Monnet X (2010). Early administration of norepinephrine increases cardiac preload and cardiac output in septic patients with life-threatening hypotension. Crit Care Lond Engl.

[32] Dünser MW, Hasibeder WR (2009). Sympathetic overstimulation during critical illness: adverse effects of adrenergic stress. J Intensive Care Med.

[33] Rivers E, Nguyen B, Havstad S, The Early Goal-Directed Therapy Collaborative Group (2001). Early goal-directed therapy in the treatment of severe sepsis and septic shock. N Engl J Med.

[34] Pruinelli L, Westra BL, Yadav P (2018). Delay Within the 3-hour Surviving Sepsis Campaign guideline on mortality for patients with severe sepsis and septic shock. Crit Care Med.

[35] Yearly DM, Kellum JA, ProCESS Investigators (2014). A randomized trial of protocol-based care for early septic shock. N Engl J Med.

[36] Peake SL, ARISE Investigators, Group ACT (2014). Goal-directed resuscitation for patients with early septic shock. N Engl J Med.

[37] Mouncey PR, Osborn TM, Power GS (2015). Trial of early, goal-directed resuscitation for septic shock. N Engl J Med.

[38] Rowan KM, Angus DC, PRISM Investigators (2017). Early, goal-directed therapy for septic shock—a patient-level meta-analysis. N Engl J Med.

[39] Bai X, Yu W, Ji W (2014). Early versus delayed administration of norepinephrine in patients with septic shock. Crit Care Lond Engl.

[40] Marik PE, Cavallazzi R, Vasu T (2009). Dynamic changes in arterial waveform derived variables and fluid responsiveness in mechanically ventilated patients: a systematic review of the literature. Crit Care Med.

[41] Gordon AC, Mason AJ, Thirunavukkarasu N, The VANISH Investigators (2016). Effect of early vasopressin vs norepinephrine on kidney failure in patients with septic shock: the VANISH randomized clinical trial. JAMA.

[42] Cecconi M, Hofer C, Teboul JL, The FENICE Investigators, ESICM Trial Group (2015). Fluid challenges in intensive care: the FENICE study: a global inception cohort study. Intensive Care Med.

[43] Marik PE, Linde-Zwirble WT, Bittner EA (2017). Fluid administration in severe sepsis and septic shock, patterns and outcomes: an analysis of a large national database. Intensive Care Med.

[44] Boyd JH, Forbes J, Nakada TA (2011). Fluid resuscitation in septic shock: a positive fluid balance and elevated central venous pressure are associated with increased mortality. Crit Care Med.

[45] Silversides JA, Fitzgerald E, Manickavasagam US (2018). Deresuscitation of patients with iatrogenic fluid overload is associated with reduced mortality in critical illness. Crit Care Med.

[46] Semler MW, Self WH, Wanderer JP (2018). Balanced crystalloids versus saline in critically Ill adults. N Engl J Med.

[47] Finfer S, McEvoy S, SAFE Study Investigators (2011). Impact of albumin compared to saline on organ function and mortality of patients with severe sepsis. Intensive Care Med.

[48] Caironi P, Tognoni G, Masson S, The ALBIOS Study Investigators (2014). Albumin replacement in patients with severe sepsis or septic shock. N Engl J Med.

[49] Mira JP. Facts or myths: early albumin resuscitation during septic shock (the EARSS trial) [Internet]. Berlin [cited 2013 Jun 17]. Available from: http://www.esicm.org/flashConference/2011/Berlin/10438/swf/player.swf.2011.

[50] Bansal M, Farrugia A, Balboni S (2013). Relative survival benefit and morbidity with fluids in severe sepsis—a network meta-analysis of alternative therapies. Curr Drug Saf.

[51] Xu J-Y, Chen Q-H, Xie J-F (2014). Comparison of the effects of albumin and crystalloid on mortality in adult patients with severe sepsis and septic shock: a meta-analysis of randomized clinical trials. Crit Care Lond Engl.

[52] Patel A, Laflan MA, Waheed U (2014). Randomised trials of human albumin for adults with sepsis: systematic review and meta-analysis with trial sequential analysis of all-cause mortality. BMJ.

[53] Rochwerg B, Alhazzani W, Sindi A, From the Fluids in Sepsis and Septic Shock Group (2014). Fluid resuscitation in sepsis: a systematic review and network meta-analysis. Ann Intern Med.

[54] Quinlan GJ, Martin GS, Evans TW (2005). Albumin: biochemical properties and therapeutic potential. Hepatology.

[55] Lai AT, Zeller MP, Millen T, The Canadian Critical Care Trials Group (2018). Chloride and other electrolyte concentrations in commonly available 5% albumin products. Crit Care Med.

[56] Fencl V, Jabor A, Kazda A (2000). Diagnosis of metabolic acid-base disturbances in critically ill patients. Am J Respir Crit Care Med.

[57] Perner A, Haase N, Guttormsen AB (2012). Hydroxyethyl starch 130/0.42 versus Ringer’s acetate in severe sepsis. N Engl J Med.

[58] Young P, Bailey M, Beasley R (2015). Effect of a buffered crystalloid solution vs saline on acute kidney injury among patients in the intensive care unit. The SPLIT Randomized Clinical Trial. JAMA.

[59] Rochwerg B, Alhazzani W, Gibson A, From FISSH Group (Fluids in Sepsis and Septic Shock) (2015). Fluid type and the use of renal replacement therapy in sepsis: a systematic review and network meta-analysis. Intensive Care Med.

[60] Kellum JA, Chawla LS, Keener C, ProCESS and ProGReSS-AKI Investigators (2016). The effects of alternative resuscitation strategies on acute kidney injury in patients with septic shock. Am J Respir Crit Care Med.

[61] Persichini R, Silva S, Teboul JL (2012). Effects of norepinephrine on mean systemic pressure and venous return in human septic shock. Crit Care Med.

[62] De Backer D, Creteur J, Silva E (2003). Effects of dopamine, norepinephrine, and epinephrine on the splanchnic circulation in septic shock: which is best?. Crit Care Med.

[63] MacGregor DA, Prielipp RC, Butterworth JF, James RL, Royster RL (1996). Relative efficacy and potency of beta-adrenoceptor agonists for generating cAMP in human lymphocytes. Chest.

[64] Ensinger H, Geisser W, Brinkmann A, Wachter U, Vogt J, Radermacher P, Georgieff M, Träger K (2002). Metabolic effects of norepinephrine and dobutamine in healthy volunteers. Shock.

[65] Silverman HJ, Penaranda R, Orens JB (1993). Impaired β-adrenergic receptor stimulation of cyclic adenosine monophosphate in human septic shock: association with myocardial hyporesponsiveness to catecholamines. Crit Care Med.

[66] Stolk RF, van der Poll T, Angus DC (2016). Potentially inadvertent immunomodulation: norepinephrine use in sepsis. Am J Respir Crit Care Med.

[67] Barth E, Albuszies G, Baumgart K (2007). Glucose metabolism and catecholamines. Crit Care Med.

[68] Andreis DT, Singer M (2016). Catecholamines for inflammatory shock: a Jekyll-and-Hyde conundrum. Intensive Care Med.

[69] Hartmann C, Radermacher P, Wepler M (2017). Non-hemodynamic effects of catecholamines. Shock.

[70] Dünser MW, Ruokonen E, Pettilä V (2009). Association of arterial blood pressure and vasopressor load with septic shock mortality: a post hoc analysis of a multicenter trial. Crit Care Lond Engl.

[71] Schmittinger CA, Dünser MW, Torgersen C (2013). Histologic pathologies of the myocardium in septic shock: a prospective observational study. Shock.

[72] Singer M (2007). Catecholamine treatment for shock–equally good or bad?. Lancet.

[73] Singer M, Matthay MA (2011). Clinical review: thinking outside the box—an iconoclastic view of current practice. Crit Care Lond Engl.

[74] McIntyre WF, Um KJ, Alhazzani W (2018). Association of vasopressin plus catecholamine vasopressors vs catecholamines alone with atrial fibrillation in patients with distributive shock. A systematic review and metanalysis. JAMA.

[75] Walkey AJ, Soylemez Wiener R, Ghobrial JM (2001). Incident stroke and mortality associated with new-onset atrial fibrillation in patients hospitalized with severe sepsis. JAMA.

[76] Bracht H, Calzia E, Georgieff M (2012). Inotropes and vasopressors: more than haemodynamics!. Br J Pharmacol.

[77] Russell JA, Lee T, Singer J, The Vasopressin and Septic Shock Trial (VASST) Group (2017). The septic shock 3.0 definition and trials: a Vasopressin and septic shock trial experience. Crit Care Med.

[78] Hajjar LA, Vincent JL, Barbosa Gomes Galas FR (2017). Vasopressin versus norepinephrine in patients with vasoplegic shock after cardiac surgery: the VANCS randomized controlled trial. Anesthesiology.

[79] Vincent JL, Su F (2008). Physiology and pathophysiology of the vasopressinergic system. Best Pract Res Clin Anaesthesiol.

[80] Vincent JL, De Backer D (2013). Circulatory shock. N Engl J Med.

[81] Russell JA, Vincent JL, Kjølbye AL (2017). Selepressin, a novel selective vasopressin V_1A_ agonist, is an effective substitute for norepinephrine in a phase IIa randomized, placebo-controlled trial in septic shock patients. Crit Care Lond Engl.

[82] Beesley SJ, Weber G, Sarge T (2018). Septic cardiomyopathy. Crit Care Med.

[83] Gordon AC, Perkins GD, Singer M (2016). Levosimendan for the prevention of acute organ dysfunction in sepsis. N Engl J Med.

[84] White FN, Gold EM, Vaughn DL (1967). Renin-aldosterone system in endotoxin shock in the dog. Am J Physiol.

[85] Levy B, Fritz C, Tahon E (2018). Vasoplegia treatments: the past, the present, and the future. Crit Care Lond Engl.

[86] Khanna A, English SW, Wang XS, The ATHOS-3 Investigators (2017). Angiotensin II for the Treatment of Vasodilatory Shock. N Engl J Med.

[87] Lira A, Pinsky MR (2014). Should β-blockers be used in septic shock?. Crit Care Lond Engl.

[88] Morelli A, Ertmer C, Westphal M (2013). Effect of heart rate control with esmolol on hemodynamic and clinical outcomes in patients with septic shock: a randomized clinical trial. JAMA.

[89] Coquerel D, Sainsily X, Dumont L (2018). The apelinergic system as an alternative to catecholamines in low-output septic shock. Crit Care Lond Engl.

[90] Bollaert PE, Charpentier C, Levy B, Debouverie M, Audibert G, Larcan A (1998). Reversal of late septic shock with supraphysiologic doses of hydrocortisone. Crit Care Med.

[91] Schelling G, Stoll C, Kapfhammer HP (1999). The effect of stress doses of hydrocortisone during septic shock on posttraumatic stress disorder and health-related quality of life in survivors. Crit Care Med.

[92] Sprung CL, Annane D, Keh D (2008). Hydrocortisone therapy for patients with septic shock. N Engl J Med.

[93] Venkatesh B, Finfer S, Cohen J (2018). Adjunctive glucocorticoid therapy in patients with septic shock. N Engl J Med.

[94] Annane D, Renault A, Brun-Buisson C (2018). Hydrocortisone plus fludrocortisone for adults with septic shock. N Engl J Med.

[95] Keh D, Boehnke T, Weber-Cartens S (2003). Immunologic and hemodynamic effects of “low-dose” hydrocortisone in septic shock: a double-blind, randomized, placebo-controlled, crossover study. Am J Respir Crit Care Med.

[96] Keh D, Trips E, Marx G (2016). Effect of hydrocortisone on development of shock among patients with severe sepsis: the HYPRESS randomized clinical trial. JAMA.

[97] Russell JA, Walley KR, Gordon AC (2009). Interaction of vasopressin infusion, corticosteroid treatment, and mortality of septic shock. Crit Care Med.

